# Reiter syndrome following protracted symptoms of Cyclospora infection.

**DOI:** 10.3201/eid0706.010635

**Published:** 2001

**Authors:** V S Sloan


by widespread drug usage, and we emphasize that targeted treatment has the potential advantage of prolonging the useful lifespan of a drug such as praziquantel.

The conclusion of our modeling analysis is that there may be only a 7- to 10-year period during which control projects will  consistent, drug-mediated reductions in worm burden. It is essential, therefore, that planners anticipate eventual drug failure and incorporate, as part of an integrated infection-management system, nondrug interventions that will prolong drug usefulness. Prevention of transmission and not just development of newer drugs will finally provide the best form of "therapy."

**C. H. King,* J.H. Ouma,† and E.M. Muchiri†**
*Case Western Reserve University School of Medicine Cleveland, Ohio, USA; and †Ministry Of Health, Nairobi, Kenya

1. King CH, Muchiri EM, Ouma JH. Evidence against rapid emergence of praziquantel resistance in *Schistosoma haematobium*, Kenya. Emerg Infect Dis 2000;6:585-94.
2. Jordan P, Webbe G. Epidemiology. In: Jordan P, Webbe G, Sturrock RF, editors. Human schistosomiasis. Wallingford, UK: CAB International; 1993. p. 87-158.
3. van Wyk JA. Refugia—overlooked as perhaps the most potent factor concerning the development of anthelmintic resistance. Onderstepoort J Vet Res 2001;68:55-67.

### Reiter Syndrome Following Protracted Symptoms of *Cyclospora* Infection

**To the Editor:** I read with interest and some dismay the report by Connor et al. on Reiter syndrome following protracted symptoms of *Cyclospora* infection (1). Wallace and Weisman summarized quite eloquently the history of "Reiter's syndrome" (2). It is now well documented that the syndrome had been described several hundred years before Reiter's publication. More importantly, Hans Reiter was a war criminal, having participated in or supervised medical "experiments" conducted on concentration camp inmates by the Nazis. Wallace and Weisman suggest "Reiter does not deserve eponymous distinction. The disorder should be renamed 'reactive cutaneo-arthropathy,' or 'reactive arthritis' syndrome." I agree with this proposal, have made it my practice, and urge this journal and my colleagues to do the same.

**Victor S. Sloan**
Robert Wood Johnson Medical School
New Brunswick, New Jersey, USA

### References

1. Connor BA, Johnson E, Soave R. Reiter syndrome following protracted symptoms of *Cyclospora* infection. Emerg Infect Dis 2001;7:453-4.
2. Wallace DJ, Weisman M. Should a war criminal be rewarded with eponymous distinction? The double life of Hans Reiter (1881-1969). J Clin Rheumatol 2000;6:49-54.

